# The Lipopolysaccharide from *Capnocytophaga canimorsus* Reveals an Unexpected Role of the Core-Oligosaccharide in MD-2 Binding

**DOI:** 10.1371/journal.ppat.1002667

**Published:** 2012-05-03

**Authors:** Simon Ittig, Buko Lindner, Marco Stenta, Pablo Manfredi, Evelina Zdorovenko, Yuriy A. Knirel, Matteo dal Peraro, Guy R. Cornelis, Ulrich Zähringer

**Affiliations:** 1 Biozentrum der Universität Basel, Basel, Switzerland; 2 Division of Immunochemistry, Research Center Borstel, Leibniz-Center for Medicine and Biosciences, Borstel, Germany; 3 Laboratory for Biomolecular Modeling, Institute of Bioengineering, School of Life Sciences, Swiss Federal Institute of Technology, EPF Lausanne, Switzerland; 4 N. D. Zelinsky Institute of Organic Chemistry, Russian Academy of Sciences, Moscow, Russia; University of Massachusetts Medical School, United States of America

## Abstract

*Capnocytophaga canimorsus* is a usual member of dog's mouths flora that causes rare but dramatic human infections after dog bites. We determined the structure of *C. canimorsus* lipid A. The main features are that it is penta-acylated and composed of a “hybrid backbone” lacking the 4′ phosphate and having a 1 phosphoethanolamine (*P*-Etn) at 2-amino-2-deoxy-d-glucose (GlcN). *C. canimorsus* LPS was 100 fold less endotoxic than *Escherichia coli* LPS. Surprisingly, *C. canimorsus* lipid A was 20,000 fold less endotoxic than the *C. canimorsus* lipid A-core. This represents the first example in which the core-oligosaccharide dramatically increases endotoxicity of a low endotoxic lipid A. The binding to human myeloid differentiation factor 2 (MD-2) was dramatically increased upon presence of the LPS core on the lipid A, explaining the difference in endotoxicity. Interaction of MD-2, cluster of differentiation antigen 14 (CD14) or LPS-binding protein (LBP) with the negative charge in the 3-deoxy-d-*manno*-oct-2-ulosonic acid (Kdo) of the core might be needed to form the MD-2 – lipid A complex in case the 4′ phosphate is not present.

## Author Summary

*Capnocytophaga canimorsus*, a commensal bacterium in dog's mouths, causes rare but dramatic infections in humans that have been bitten by dogs. The disease often begins with mild symptoms but progresses to severe septicemia. The lipopolysaccharide (LPS), composed of lipid A, core and O-antigen, is one of the most pro-inflammatory bacterial compounds. The activity of the LPS has so far been attributed to the lipid A moiety. We present here the structure of *C. canimorsus* lipid A, which shows several features typical for low-inflammatory lipid A. Surprisingly, this lipid A, when attached to the core-oligosaccharide was far more pro-inflammatory than lipid A alone, indicating that in this case the core-oligosaccharide is able to contribute significantly to endotoxicity. Our further work suggests that a negative charge in the LPS-core can compensate the lack of such a charge in the lipid A and that this charge is needed not for stabilization of the final complex with its receptor but in the process of forming it. Overall the properties of the lipid A-core may explain how this bacterium first escapes the innate immune system, but nevertheless can cause a shock at the septic stage.

## Introduction

*Capnocytophaga canimorsus*, a usual member of dog's mouths flora [1] was discovered in 1976 [2] in patients who underwent dramatic infections after having been bitten, scratched or simply licked by a dog. The most common syndrome is sepsis, sometimes accompanied by peripheral intravascular coagulation and septic shock [3]. *C. canimorsus* is a Gram-negative rod belonging to the family of *Flavobacteriaceae* in the phylum *Bacteroidetes* [4], [5]. Human infections occur, worldwide, with an approximate frequency of one per million inhabitants per year [6].

*C. canimorsus* are able to escape complement killing and phagocytosis by human polymorphonuclear leukocytes and macrophages [7], [8]. Whole bacteria are also poor agonists of Toll-like receptor (TLR) 4, which results in a lack of release of pro-inflammatory cytokines by macrophages [9]. In addition to these “passive” features, *C. canimorsus* have been shown to harvest glycan moieties from glycoproteins at the surface of animal cells, including phagocytes [10], [11], [12], in addition they also deglycosylate human IgG [12].

One of the most pro-inflammatory bacterial compounds is the lipopolysaccharide (LPS, endotoxin) [13], consisting of three domains: lipid A, the core-oligosaccharide and the O-polysaccharide (O-antigen). As a potent activator of the innate immune system, LPS can induce endotoxic shock in patients suffering from septicemia. Recognition of LPS by the host occurs via the TLR4/MD-2/CD14 receptor complex [14], [15], [16], at which two proteins, CD14 and LBP, have been shown to enhance the response to LPS by transporting single LPS molecules [17], [18], [19], [20]. It has been shown that the lipid A moiety of the LPS is sufficient for TLR4 binding and stimulation [21], [22]. The interaction of lipid A and its receptor was unraveled by x-ray crystallography pioneering studies of complexes between MD-2 and the lipid A analog Eritoran [23] or lipid IV_A_ [24]. The identification of the binding sites of lipid A to MD-2 and also to the Leucine-rich repeat (LRR)-domains of TLR4 [21] is a landmark achievement that enables a deeper understanding of the structure-function relationship between LPS/lipid A and its receptors. According to these data, the 1 and 4′ phosphates of the lipid A backbone, which form charge interactions with TLR4 and MD-2, are the key elements for receptor activation [21], [25], even though for some of the interactions conflicting data have been reported [26]. It was further shown that the β-hydroxymyristate chain at position 2 forms hydrogen bonds and hydrophobic interactions with TLR4. At present, there is no evidence that the LPS-core plays any major role in binding to TLR4; only a 10- to 100-fold difference in endotoxicity of lipid A and LPS has been reported for *E. coli*, *Porphyromonas gingivalis* or *Proteus mirabilis* [27], [28], but these small differences have been attributed to changes in solubility, even if solid experimental proof is lacking. The core-oligosaccharide has so far never been shown to alter TLR4/MD-2 binding of a specific lipid A, only slight changes in MD-2 binding have been reported [29].

In this work, we investigated the lipid A structure of *C. canimorsus* in order to clarify its contribution to the septicemia and shock provoked by these bacteria. Very few lipid A structures have actually been solved in the *Cytophaga/Flavobacterium* group, with the exception of the lipid A from *Elizabethkingia meningoseptica* (former *Flavobacterium meningosepticum*) [30]. Already some time ago, the acyl chains present in the LPS of *Cytophaga* bacteria have been identified as [13-Me-14:0 (*i*15:0), 13-Me-14:0(3-OH)(*i*15:0(3-OH), 16:0(3-OH) and 15-Me-16:0(3-OH) (*i*17:0(3-OH)] [31], whereat *i*15:0 is *iso*-pentadecanoic acid (13-methyltetradecanoic acid, 13Me-14:0), *i*15:0(3-OH) represents *iso*-(*R*)-3-hydroxypentadecanoic acid [(*R*)-3-hydroxy-13-methyltetradecanoic acid, 13Me-14:0(3-OH)]; 16:0(3-OH) is (*R*)-3-hydroxyhexadecanoic acid and *i*17:0(3-OH) represents *iso*-(*R*)-3-hydroxyheptanoic acid [(*R*)-3-hydroxy-15-methylhexanoic acid, 15-Me-16:0(3-OH)]. Here we show that lipid A of *C. canimorsus* consists of the penta-acylated hybrid backbone 2,3-diamino-2,3-dideoxy-d-glucose (β-d-GlcN3N′) linked (1′→6) to α-d-GlcN where the 4′ phosphate group is missing and the 1 phosphate is linked to an ethanolamine group, forming a *P*-Etn. Not unexpectedly, this lipid A was of very low endotoxicity but, surprisingly, when bound to the core [lipid A-core (LA-core)] it became 20,000 fold more endotoxic. In agreement with this observation, we show that the LPS core promotes the binding of *C. canimorsus* lipid A to MD-2. This is the first example of a core-oligosaccharide dramatically changing the endotoxicity of lipid A, in which the carboxy group of Kdo probably takes over the function of ionic binding of the missing 4′ phosphate in the lipid A.

## Results

### Compositional analyses of lipid A

GlcN and GlcN3N were found in a ratio of approx. 2∶1 (Table 1). Based on the notion that by gas-liquid chromatography (GLC) analysis synthetic GlcN3N expressed a response factor of about 50% when compared with GlcN (or Galactosamine (GalN) as internal standard), it was inferred that GlcN and GlcN3N are present in equimolar amounts in the lipid A backbone, suggesting the presence of a “hybrid backbone” in *C. canimorsus* lipid A (Table 1). Total fatty acid analysis revealed *i*15:0, *i*15:0(3-OH), 16:0(3-OH), and *i*17:0(3-OH) in a molar ratio of approximately 1∶1∶1∶2 in lipid A preparations. Analysis of ester-bound acyl chains indicated the presence of *i*15:0 and *i*15:0(3-OH) in approximately equimolar amounts, indicating that one 16:0(3-OH) and two *i*17:0(3-OH) residues are primary acyl chains *N*-linked to the lipid A backbone (Table 1). This result suggests a penta-acylated lipid A species.

**Table 1:** Compositional analysis data of the purified lipid A of *C. canimorsus* 5 wild type.

Component	nmol/mg	mol/mol GlcN
**Sugars**		
GlcN3N^a^^,^ c ^,^ *	167	0.5
GlcN^a^^,^ c	358	1.0
**Polar substituents**		
*P* b	468	1.3
Etn-*P* c	ND	-
Etn^c^	ND	-
**Fatty acids** a		
*i*15:0	278	0.8
*i*15:0(3-OH)	416	1.2
16:0(3-OH)	417	1.2
*i*17:0(3-OH)	709	2.0

a GLC-MS data,

b Photometric assay,

c HPLC (Pico-tag).

*: Per-*O*-acetylated GlcN3N-ol can only be quantified by GLC analysis by approx. 50% compared to GlcNAc-ol, as determined by synthetic reference compound.

### High-performance liquid chromatography (HPLC) and mass spectrometry (MS) analyses of lipid A

The reversed phase HPLC profile of the lipid A sample is shown in Fig. S1. Peak 2 expressed a molecular ion at *m/z* 1716.30, which is in excellent agreement with a lipid A containing *i*15:0, *i*15:0(3-OH), 16:0(3-OH), and two moles of *i*17:0(3-OH) attached to the lipid A backbone (GlcN3N-GlcN), which also carries one *P*-Etn residue. The second major fraction (peak 5) at *m/z* 1594.29 was compatible with lipid A lacking the *P*-Etn. Based on peak intensities (peaks 2 and 5) about 40% of the *P*-Etn was liberated, most likely from the lipid A under the hydrolysis conditions used (Fig. S1).

All lipid A fractions investigated expressed a certain heterogeneity with respect the chain length of acyl chains (-CH_2_- groups), as all MS showed peak “clusters” differing by 14 u, thus suggesting acyl chain heterogeneity (Table 2, Fig. S2). Combined GLC/mass spectrometry (GLC-MS) analysis of the acyl chains revealed that the mass difference of Δ*m/z* = 14 u was not due to the exchange of one single, prominent shorter acyl chain [e.g. 16:0(3-OH)→*i*15:0(3-OH)]. Instead, the lipid A showed a certain structural “fuzziness” with respect to the size and position of the individual acyl chains, which, according to this finding, appeared to be statistically distributed over all positions with no specific structural variation.

**Table 2:** ESI-MS analysis of lipid A fractions obtained by reversed phase HPLC shown in Fig. S1.

Peak No.	Retention time in min	Yield in mg (%)	Mol. Mass of the major peak
1a	118.5	0.13 (5.8)	1660.235
1b	119.7	0.18 (8.3)	1674.265
**2**	**124.3**	**0.66 (25.5)**	**1716.300**
3	127.3	0.06 (2.8)	1716.301
**5**	**131.9**	**0.55 (24.2)**	**1594.292**
6	∼133	0.19 (8.8)	1608.306
6′	134.6	0.09 (4)	1589.266
6′			1594.290
Applied:	2.1 mg		
Total yield:	1.86 mg (88.6%)		

The major peaks shown in bold at *m/z* 1716.30 (peak 2) and *m/z* 1594.29 (peak 5) represent the lipid A and the lipid A lacking the *P*-Etn group at C-1 of GlcN.

The ESI-MS data of the wt strain shown in Table 2 indicated identical mass at *m/z* 1716.30 for peaks 2 and 3. As these lipid A fractions differed in their retention time, we conclude that they represent different structural isomers as they could be baseline-separated by HPLC. This HPLC analysis in combination with ESI-MS data thus shows that structural heterogeneity might not be solely related to the chain length of one acyl chain, but also to its position within the lipid A backbone.

In order to allocate the type of the hybrid lipid A backbone, the acyl chain distribution over the lipid A backbone, and the attachment side of the *P*-Etn, electrospray-ionization Fourier transform ion-cyclotron resonance (ESI FT-ICR) MS/MS in the positive mode was run [32]. The triethylammonium salt of HPLC purified lipid A at *m/z* 1820.40 was selected as precursor ion (Fig. S3). Infrared multiphoton dissociation (IRMPD)-MS/MS generated one abundant characteristic B-fragment oxonium-ion of the non-reducing end at *m/z* 907.77, which is in excellent agreement with the mass value calculated for GlcN3N with *i*15:0, 16:0(3-OH), and *i*17:0(3-OH) attached (*m/z* 907.77). This fragmentation pattern also showed that *P*-Etn is attached at the reducing end - most likely at position C-1. Thus the lipid A in *C. canimorsus* is penta-acylated with an acylation pattern of three being attached to the “non-reducing” GlcN3N′ and two to the reducing GlcN sugar (3+2) in the lipid A hybrid backbone.

### Nuclear magnetic resonance (NMR) analysis of lipid A

**Figure 1:**
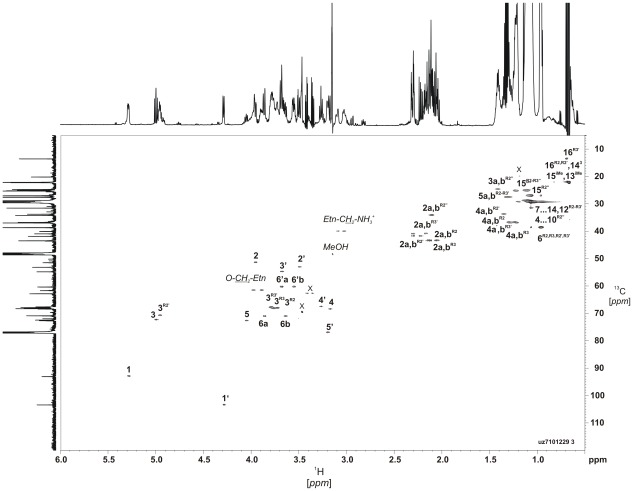
NMR analysis of the lipid A from *C. canimorsus* wild type. ^1^H,^13^C-HSQC spectrum (700 MHz) of lipid A in chloroform-methanol-water (20∶10∶1, v/v/v) at 27°C. The corresponding parts of the ^13^C and ^1^H NMR spectra are displayed along the F1 and F2 axes, respectively. Numerals refer to atoms in sugar and acyl chain residues denoted by letters as shown in Supplementary Table 1 and Fig. S2. Signals from an unidentified contaminating lipid are indicated by X.

**Figure 2:**
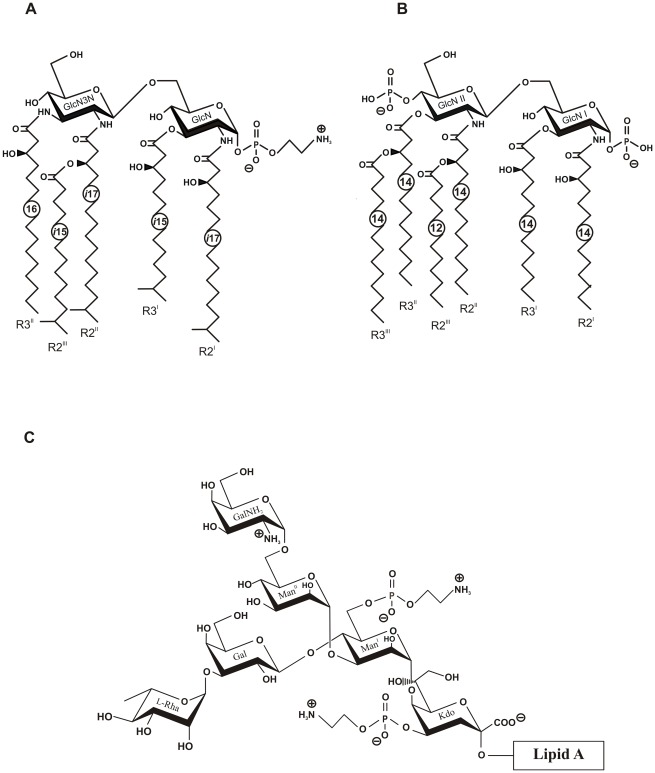
Structures of *C. canimorsus* lipid A, *E. coli* lipid A and core-oligosaccharide of *C. canimorsus* attached to the lipid A. (A) *C. canimorsus* lipid A consists of a β-(1′→6)-linked GlcN3N′-GlcN disaccharide, to which 3-hydroxy-15-methylhexadecanoic acid, 3-hydroxy-13-methyltetradecanoic acid, 3-*O*-(13-methyltetradecanoyl)-15-methylhexadecanoic acid, and 3-hydroxyhexadecanoic acid are attached at positions 2, 3, 2′, and 3′, respectively. The disscharide carries a positively charged ethanolamine at the 1 phosphate and lacks a 4′ phosphate. (B) Structure of *E. coli* hexa-acylated lipid A. (C) *C. canimorsus* LPS core features only one Kdo, to which a phosphoethanolamine (*P*-Etn) is attached. The only net negative charge present is from the carboxy group of the Kdo. The inner core continues with Man to which another a *P*-Etn is attached. The outer core consists of Gal and l-Rhamnose (l-Rha), to which the O-antigen is attached (U. Zähringer, unpublished results).

The lipid A was studied further by high-field NMR spectroscopy using correlation spectroscopy (COSY), total correlation spectroscopy (TOCSY), rotating-frame nuclear Overhauser effect spectroscopy (ROESY), ^1^H,^13^C-heteronuclear single-quantum coherence (HSQC), ^1^H,^31^P-heteronuclear multiple-quantum coherence (HMQC), and ^1^H,^31^P-HMQC-TOCSY experiments. The results are depicted in the supplement (Table S1). The ^1^H,^13^C-HSQC spectrum (Fig. 1) showed two H-1,C-1 cross-peaks at δ 4.28/103.4 and 5.29/92.8 for GlcN3N′ and GlcN, which were distinguished by correlations between protons at nitrogen-bearing carbons and the corresponding carbons (C-2′ and C-3′ of GlcN3N′ and C-2 of GlcN, at δ 52.9, 54.6, and 51.4, respectively). ^3^ *J*_1,2_ coupling constants of 8.0 and 2.9 Hz for the H-1 signals at δ 4.28 and 5.29, were determined from the ^1^H NMR spectrum and showed that GlcN3N is β- and GlcN α-linked. The H-1 signal of α-GlcN was additionally split due to coupling to phosphorus (^2^ *J*_1,P_ 7.9 Hz), thus indicating that α-GlcN is phosphorylated with *P*-Etn and β-GlcN3N′ represents the “non-reducing” end of the lipid A backbone. The β1′→6-linkage between the two amino sugars was evident from strong cross-peaks of H-1′ of GlcN3N′ with protons H-6a′,6b′ of GlcN at δ 3.64 and 3.87 in the ROESY spectrum. The location of the *P*-Etn residue at position 1 of α-GlcN was further confirmed by ^1^H,^31^P-HMQC and ^1^H,^31^P-HMQC-TOCSY (Fig. S4) as well as ROESY experiments, which showed correlations between H-1 of GlcN at δ 5.29 and H-1a,1b of ethanolamine (Etn) at δ 3.91 and 3.98. In accordance with the 1′→6 linkage and the position of GlcN3N at the “non-reducing end”, the ^13^C NMR spectrum (Table S1) displayed a typical down-field displacement by ∼10 ppm for C-6 of the 6-substituted GlcN (δ 71.0; compared with δ 60.0 for C-6 of GlcN3N′, which is non-substituted in the free lipid A). The acylation pattern was confirmed by ^1^H,^13^C-HSQC spectroscopy (Fig. 1), which showed only one characteristic downfield shift due to a deshielding effect for the *i*17:0[3-*O*(*i*15:0)] R2′ i.e. the H-3/C-3 R2′ cross-peak at δ 4.95/70.7. This finding indicated that only the OH-group of *i*17-0(3-OH) is acylated giving rise to an acyloxyacyl residue [*i*17:0-3-*O*(*i*15:0)] showing a 3+2 type acyl chain distribution in the penta-acylated lipid A, which is in good agreement with the MS data (Figs. S2 and S3). Taking together the data of the chemical studies defines the structure of the lipid A of *C. canimorsus* shown in Fig. 2 A. The structure of *E. coli* hexa-acylated lipid A is depicted for comparison (Fig. 2 B). The *E. coli* lipid A consists of a β-(1′→6)-linked GlcN disaccharide that is phosphorylated at positions 1 and 4′ and carries four (*R*)-3-hydroxymyristate chains (at positions 2′, 3′, 2 and 3). The R2′ and R3′ 3-hydroxylated acyl groups in GlcN′ are further esterified with laurate and myristate, respectively [22].

### *C. canimorsus* LPS core features only one Kdo

The structure of *C. canimorsus* LA-core is depicted in Fig. 2 C and its structural analysis will be described elsewhere (Zähringer et al., manuscript in preparation). The *C. canimorsus* LPS core features only one Kdo, to which a *P*-Etn is attached in position 4. Usually, mono-Kdo LPS-core have a phosphate attached to the Kdo at that position. Thus, the only net negative charge in this core oligosaccharide originates from the carboxy-group of the Kdo. The inner core continues with two mannoses (Man) to which another *P*-Etn is attached in position 6 of Man^I^ residue in the core oligosaccharide. The outer core consists of Galactose (Gal) and l-Rhamnose (to which the O-antigen is attached). A positively charged Galactosamine (GalN) residue is linked to the (second) Man^II^ residue in position 6 (U. Zähringer, unpublished results).

### The structure identified matches the *C. canimorsus* genome

**Figure 3:**
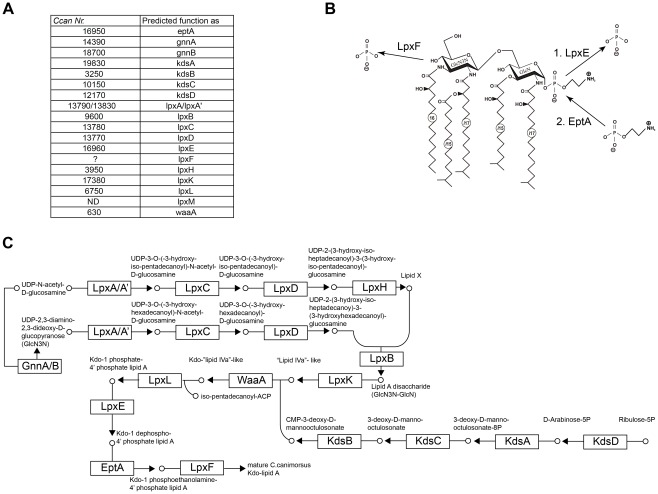
Biosynthesis of *C. canimorsus* lipid A-Kdo. (A) Alphabetic list of enzymes required and the corresponding gene codes in the *C. canimorsus* 5 genome are listed. (B) Proposed enzymatic modification on lipid A by LpxF, LpxE and EptA. (C) Single steps in the biosynthesis of *C. canimorsus* lipid A-Kdo (adapted from KEGG map00540).

*E. coli* lipid A biosynthesis has been unravelled in detail [22], [33]. Analyzing the genome of *C. canimorsus* 5 [5], we identified the genes required for the synthesis of lipid A-Kdo [33]. Only *lpxA*, *lpxA′*, *lpxC* and *lpxD* seem to cluster in one operon, the other genes are dispersed (Fig. 3 A). The difference in acylation of the 3′ and 3 position and the hybrid backbone of the lipid A consisting of a β-1′,6-linked GlcN3N′-GlcN disaccharide, suggests that two *lpxA* genes might be present in *C. canimorsus* and indeed two *lpxA* genes were identified (termed *lpxA* and *lpxA′*) in the *C. canimorsus* 5 genome (Fig. 3 A). In *Acidithiobacillus ferrooxidans* GnnA and GnnB are responsible for the biosynthesis of GlcN3N [34]. Based on the sequences of *A. ferrooxidans*, *gnnA* and *gnnB* could be identified in the genome of *C. canimorsus* (Fig. 3 A). In the biosynthetic pathway of *E. coli* lipid A, enzyme LpxM adds the acyloxyacyl-residue [14:0-3-*O*(14:0)] representing the 6^th^ acyl chain [22]. In good agreement with the penta-acylation of lipid A in *C. canimorsus* 5 was our finding that *lpxM* could not be identified in the genome (Fig. 3 A). *C. canimorsus* LPS core features only one Kdo, suggesting a mono-functional Kdo transferase (WaaA/KdtA) or a Kdo hydrolase two-protein complex (KdoH1/2) as in *Helicobacter pylori* or *Francisella novicida* [35], [36]. Searches with KdoH1/2 did not hit any gene in the *C. canimorsus* 5 genome. Therefore, *C. canimorsus* possesses either a mono-functional WaaA or a KdoH1/2 complex without significant sequence similarity to known Kdo hydrolases. We have further investigated the enzymes leading to the addition of an Etn at the 1 phosphate of lipid A. In *H. pylori*, the addition of a *P*-Etn at 1 position has been proposed to result from a two-step mechanism [37]. In a first step the 1 phosphate is removed by a phosphatase (LpxE), and subsequently a *P*-Etn-transferase (EptA or PmrC, YjdB) adds a *P*-Etn to the 1 position of lipid A [37] (Fig. 3 B). In *H. pylori lpxE* and *eptA* are encoded by one operon (Hp0021-Hp0022). *C. canimorsus eptA* was annotated as *Ccan* 16950. Search for a lipid A phosphatase were based on *lpxE* and/or *lpxF* sequences from *P. gingivalis* [38], *F. novicida* [39], *Rhizobium etli* [40] *H. pylori* [37], [41] and on all available *Bacteroidetes*-group *pgpB* sequences. Three *lpxE/F* candidates have been found in the *C. canimorsus 5* genome (*Ccan*16960, *Ccan*14540 and *Ccan*6070). All candidates were deleted and the mutated bacteria were tested for endotoxicity. Only deletion of *Ccan16960* affected endotoxicity (data not shown). Interestingly, *Ccan16960* is located within the same operon as *eptA* and the two genes overlap by 20 bp. Following the operon organisation of *H. pylori*, *Ccan*16960 has been annotated as *lpxE*. The predicted function of *lpxE* and *eptA* was validated by KO and analysis of the resulting phenotype (Ittig et al., manuscript in preparation).

The presence of the 4′ kinase LpxK and the absence of a 4′ phosphate leads to the assumption of the presence of a 4′ phosphatase, LpxF. Several candidate genes were identified (besides *lpxE*: *Ccan 14540* and *Ccan6070*) and deleted but they had to be ruled out, as no deletion did affect the endotoxic activity (data not shown), thus, we lack annotation of *lpxF*. The proposed complete biosynthesis of *C. canimorsus* lipid A-Kdo is depicted in Fig. 3 C, starting from UDP-N-acetyl-d-glucosamine and ribulose-5 phosphate.

### *C. canimorsus* LPS is 100-fold less endotoxic than *E. coli* O111 LPS

**Figure 4:**
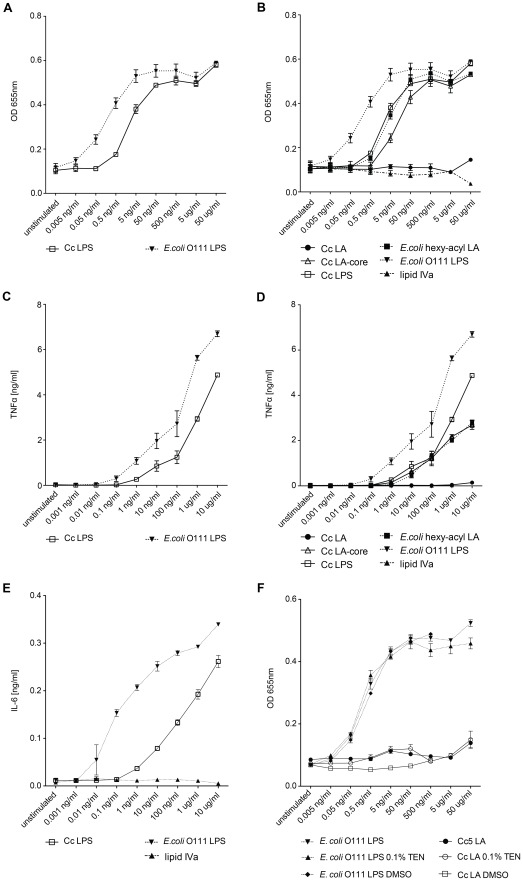
Endotoxic activity of *C. canimorsus* (Cc) LPS, lipid A (LA) or lipid A-core (LA-core) and contribution of the LPS core to endotoxicity. (A–B) Dose-response curve of purified lipid A, LA-core or LPS. Samples were assayed for TLR4 dependent NFκB activation with HEKBlue human TLR4 cells. (C–D) Purified lipid A, LA-core or LPS samples were assayed for induction of TNFα release by human THP-1 macrophages. (E) Purified LPS samples or lipid IV_A_ were assayed for induction of IL-6 release by canine DH82 macrophages. (F) Dose-response curve of NFκB activation by lipid A or LPS. Purified lipid A or LPS samples were assayed for TLR4-dependent NFκB activation with HEKBlue human TLR4 cells. The *C. canimorsus* lipid A stock solution was pretreated with either 0.1% TEN or 50% DMSO and sonication to increase its solubility in water/buffer. Identical concentrations of DMSO or TEN were added to *E. coli* O111 LPS as a control. Data were combined from n = 3 independent experiments, error bars indicated are standard error of the mean.

The endotoxic activity of wt *C. canimorsus* 5 LPS (S-form) was compared to the endotoxic activity of *E. coli* O111 LPS using three different approaches: (i) Purified LPS samples were assayed for TLR4 dependent NFκB activation with HEK293 cells overexpressing human TLR4/MD-2/CD14 and a secreted reporter protein (HEKBlue human TLR4 cell line), (ii) purified LPS samples were assayed for induction of TNFα release by human THP-1 macrophages, (iii) purified LPS samples were tested for stimulation of IL-6 release by canine DH82 macrophages. In the two assays involving human TLR4 (Fig. 4 A and Fig. 4 C) *C. canimorsus* LPS appeared to be about 100 fold less endotoxic than *E. coli* O111 LPS (both S-form LPS). In contrast to human macrophages, where *C. canimorsus* LPS was found 10–100 fold less endotoxic than *E. coli* O111 LPS (Fig. 4 B), for canine macrophages the difference in endotoxicity of the two LPS was around 1000 fold (Fig. 4 E). In addition, lipid IV_A_ seems not to be an agonist of canine TLR4 as is the case for murine TLR4 [42].

### *C. canimorsus* lipid A and LA-core exhibit striking difference in endotoxicity

Generally, the lipid A part of a LPS is considered as sufficient to trigger full TLR4 activation. Minor differences between lipid A and LPS or LA-core have so far been attributed to differential bioavailability/solubility in water even if solid experimental proof is lacking. We have, therefore, examined the endotoxic activity of *C. canimorsus* lipid A, LA-core and LPS using the HEKBlue hTLR4 cell line and the TNFα release by human THP-1 macrophages. LPS and LA-core exhibited an endotoxicity in the same range, whereas the LPS was less than 10-fold more endotoxic than the LA-core (Fig. 4 B and Fig. 4 D). In contrast, *C. canimorsus* lipid A appeared to be absolutely non-stimulatory up to 5 µg/ml (Fig. 4 B and Fig. 4 D), around 20,000-fold less active than the LA-core and 200,000-fold less active than LPS on a weight basis (ng/ml) indicating a even higher difference on a molar basis. As the *C. canimorsus* LPS and the LA-core showed similar endotoxicity, the increase in endotoxicity in comparison to the lipid A must have been raised by the contribution of the core oligosaccharide. Minor differences in endotoxicity between LPS and LA-core as the 10- to 100-fold difference observed between *E. coli* lipid A and *E. coli* O111 LPS (Fig. 4 B and Fig. 4 D) might be explained by differential bioavailability/solubility in water/buffer and by a direct contribution of the core-oligosaccharide in TLR4/MD-2 binding as suggested [21]. However, in the case of *C. canimorsus* LA-core the direct contribution of the core-oligosaccharide might be far more pronounced as in *E. coli*, since *C. canimorsus* has a lipid A lacking a net negative charge. A role of the core-oligosaccharide in providing solubility to lipid A was ruled out by the fact that no increase in endotoxicity was observed by the addition of triethylamine (TEN) or dimethyl sulfoxide (DMSO) to the *C. canimorsus* lipid A stock solution followed by sonication (see Fig. 4 F).

### *C. canimorsus* LPS core is essential for proper MD-2 binding of the lipid A

The increase in endotoxicity of the *C. canimorsus* LA-core in comparison to the lipid A must have been raised by the contribution of the core oligosaccharide (Fig. 4). The 4′ phosphate of *E. coli* lipid A is known to interact with Arg_264_ and Lys_362_ of TLR4 and Lys_58_ and Ser_118_ of MD-2 [21]. *C. canimorsus* lipid A lacks the 4′ phosphate and features only one net negative charge in the LPS core, namely the carboxylic oxygen of Kdo. Based on the known structure of *E. coli* LPS bound to TLR4/MD-2 (3FXI, [21]) we measured the interaction distances from the carboxylic oxygen of Kdo to Arg_264_ and Lys_362_ of TLR4 and to Lys_58_ and Ser_118_ of MD-2. The carboxylic oxygen of Kdo is within close distance to Arg_264_ and Lys_362_ of TLR4 and Lys_58_ and Ser_118_ of MD-2 and hence could contribute to binding to MD-2 or TLR4.

**Figure 5:**
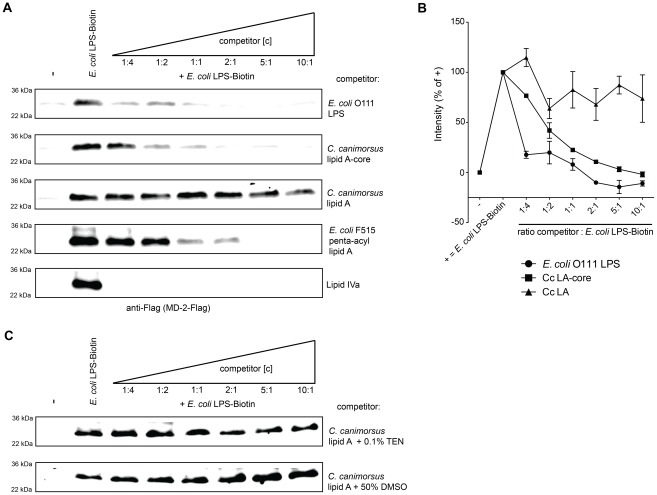
Binding to human MD-2 of *C. canimorsus* lipid A depends on the core-oligosaccharide. Soluble human MD-2 from cell culture supernatant was combined with the indicated mixture of *E. coli* LPS-Biotin and a competitor (either *C. canimorsus* lipid A, lipid A-core, *E. coli* O111 LPS, penta-acyl *E. coli* lipid A or lipid IV_A_). Biotinylated *E. coli* LPS-MD-2 complexes were purified and analyzed by non-reducing, denaturing Western blotting for presence of MD-2. (A) Untreated human MD-2 did not bind to the Strep-column (lane 1), addition of *E. coli* LPS-biotin lead to co-purification of human MD-2 (lane 2). Results shown are representative of three independent determinations. (B) Quantification of Western-blots as shown in A. Values are shown as percentage of the corresponding positive control. Data were combined from n = 3 independent experiments, error bars indicated are standard error of the mean. (C) as in (A) but the *C. canimorsus* lipid A stock solution was pretreated with either 0.1% TEN or 50% DMSO and sonication in both cases to increase its solubility in water/buffer.

To assess the ability of *C. canimorsus* lipid A or LA-core to interact with human MD-2, we monitored their ability to compete with the binding of *E. coli* LPS-Biotin to MD-2. Culture supernatants of cells producing human MD-2 were incubated with biotinylated *E. coli* O111 LPS, either alone or in combination with different concentrations of a competitor. As a source of LBP and soluble CD14, 7.5% FCS (v/v) was added. After purification of LPS based on biotin, co-purification of MD-2 was monitored by Western blotting. *C. canimorsus* LA-core abolished the copurification of MD-2 with the *E. coli* LPS-Biotin at higher concentration than the positive controls, *E. coli* O111 LPS and lipid IV_A_ but at lower concentration than unbiotinylated *E. coli* penta-acyl lipid A (Fig. 5 A and B). Lipid IV_A_ is expected to be a very potent competitor, as it has been shown to bind deeper into the MD-2 pocket and thus likely stronger to MD-2 than *E. coli* lipid A [21], [24]. These results indicate that *C. canimorsus* LA-core binds to human MD-2, likely in the same pocket as *E. coli* LPS. This experiment does not reflect the antagonistic capacity of *C. canimorsus* LA-core as even native *E. coli* O111 LPS could prevent the co-purification of human MD-2 (Fig. 5 A and B). In contrast to the LA-core, *C. canimorsus* lipid A did not significantly affect the copurification of MD-2 with *E. coli* LPS-Biotin even at high concentration (Fig. 5 A and B). Thus, *C. canimorsus* lipid A seems not to bind to human MD-2 at all or to bind to MD-2 only very weakly, in contrast to the LA-core. To rule out a major contribution of the core-oligosaccharide by providing solubility to the lipid A, the same MD-2 binding experiment has been performed with *C. canimorsus* lipid A pre-treated with DMSO or TEN and sonicated to improve solubility. These *C. canimorsus* lipid A samples did not significantly affect the copurification of MD-2 with *E. coli* LPS-Biotin even at high concentration (Fig. 5 C). We conclude from this experiment that the *C. canimorsus* LPS core promotes the interaction and binding of the lipid A to MD-2 either via direct interaction with MD-2 or via binding to LBP or CD14.

### The final complex of human MD-2 and lipid A of *C. canimorsus* would be as stable as MD-2 and lipid A of *E. coli*

**Figure 6:**
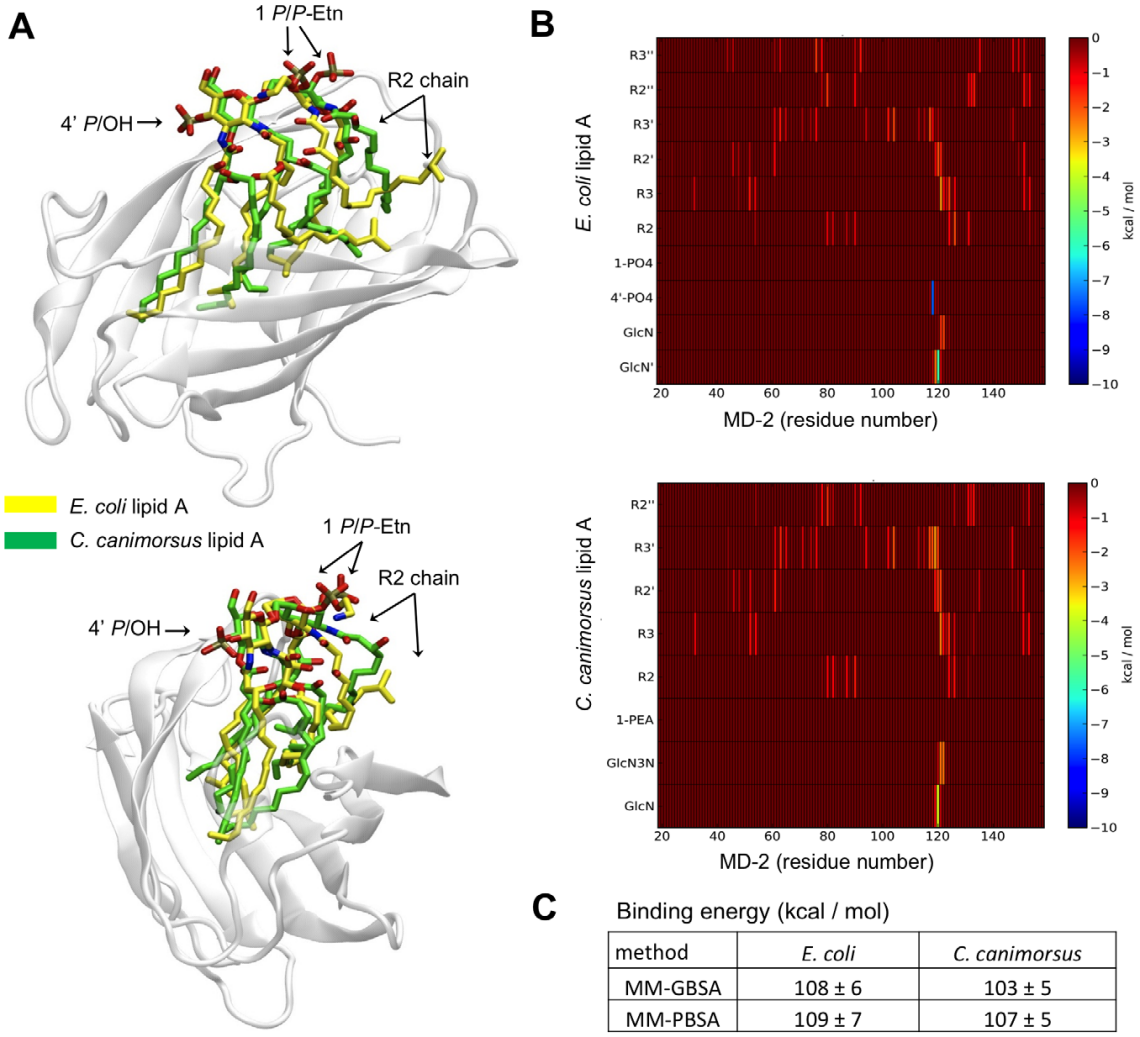
Modeled binding of *C. canimorsus* lipid A to human MD-2. (A) Front and side view of the equilibrated complexes between MD-2 (gray) and *C. canimorsus* (yellow) and *E. coli* (green) lipid A. (B) Pairwise decomposition of the global total (Van der Waals+electrostatic+solvation) binding free energy calculated at MM-GBSA level. (C) Binding energy between MD-2 and the two lipid A molecules calculated using the MM-GBSA and MM-PBSA methods on 300 snapshots extracted from two 10 ns long equilibrated NPT molecular dynamics simulations.

In order to assess the contribution of the *C. canimorsus* LPS core in binding of the lipid A to MD-2, we modelled the binding of *C. canimorsus* lipid A to human MD-2 (Fig. 6 A) and compared it to the binding of *E. coli* lipid A. Some differences between the two complexes could be observed at the level of the lipid chains after just few ns of simulation (Fig. 6 A). In both cases the R3′ and R3 chains (see Fig. 2 for nomenclature) were fully stretched and interacted with the same residues. No empty space was left by R3″ (missing in *C. canimorsus*) because the longer R2′ and R2″ chains filled the void. While in *E. coli* the R2 chain is stretched toward the inner side of the pocket, in *C. canimorsus* it was projected toward the pocket exterior, due to both i) its longer size and ii) to the presence of the bifurcated terminus of the close R2″. The R2 chain of *C. canimorsus* lipid A was thus not completely buried inside the MD-2 pocket and it was even more exposed to the surface than the hydroxymyristate chain at position 2 in *E. coli*. This probably enables the *i*17:0(3-OH) chain at position 2 to interact with TLR4, as has been reported for the R2 chain of hexa-acylated *E. coli* LPS [21]. It should be mentioned here that penta-acylated *E. coli* lipid A is endotoxically almost inactive [13], and the acyl chains might be completely buried inside MD-2. Thus *C. canimorsus* penta-acylated lipid A is expected to behave differently from penta-acylated *E. coli* lipid A due to the extended length of the acyl chains and the bulky iso-groups. Overall the arrangement of the sugar moieties with respect to the MD-2 was similar for both complexes, the only major discrepancies being the orientation of the 1-phosphoryl group (1 phosphate in *E. coli*, 1 *P*-Etn in *C. canimorsus*). The calculated binding energy for the two complexes was very similar when calculated at both MM-GBSA (molecular mechanics, the generalized Born model and solvent accessibility) and MM-PBSA (molecular mechanics, Poisson-Boltzmann solvent accessible surface area) level, being in both cases the MD-2 – *E. coli* lipid A complex slightly more stable (Fig. 6 C). To understand this trend the total binding free energy was fractionated into a list of interaction energies between each residue of MD-2 and each fragment of lipid A (Fig. 6 B), as coded in Fig. 2. Each pairwise binding free energy value has been further fractioned into its electrostatic, steric (Van der Waals), and solvation (polar and cavitation) components. For each term contributions arising from backbone and sidechain have been singled out. In both cases the GlcN′ (*E. coli*) or the GlcN3N′ (*C. canimorsus*) moieties (2′ NH group) interacted with the backbone carbonyl of Ser_120_ establishing a strong (about 4–5 kcal/mol) and persistent interaction. Favorable interactions were also observed between GlcN and residues Phe_121_ and Lys_122_. The side chain of Phe_121_ established a strong apolar interaction (Van der Waals, non-polar solvation) with the extended R3 acyl chain in both complexes. The hydrogen bond between the NH group of Ser_120_ and the carbonyl of the R3′ chain was found to be strong and persistent in both cases. Neither the 1 phosphate group (*E. coli*) nor the 1 *P*-Etn (*C. canimorsus*) established favorable interactions with MD-2, whereas the 4′ phosphate group (missing in *C. canimorsus*) could be accounted for the slightly greater stability of the MD-2 *E. coli* lipid A complex, due to the strong (about 7.5 kcal/mol) interaction established with both the backbone and the sidechain of Ser_118_ (see Fig. 6 B). In summary, we found that in the final complex the arrangement of the sugar moieties with respect to the MD-2 and the calculated binding energy for the two complexes was very similar for *E. coli* lipid A and *C. canimorsus* lipid A.

### *C. canimorsus* lipid A is no antagonist of TLR4

**Figure 7:**
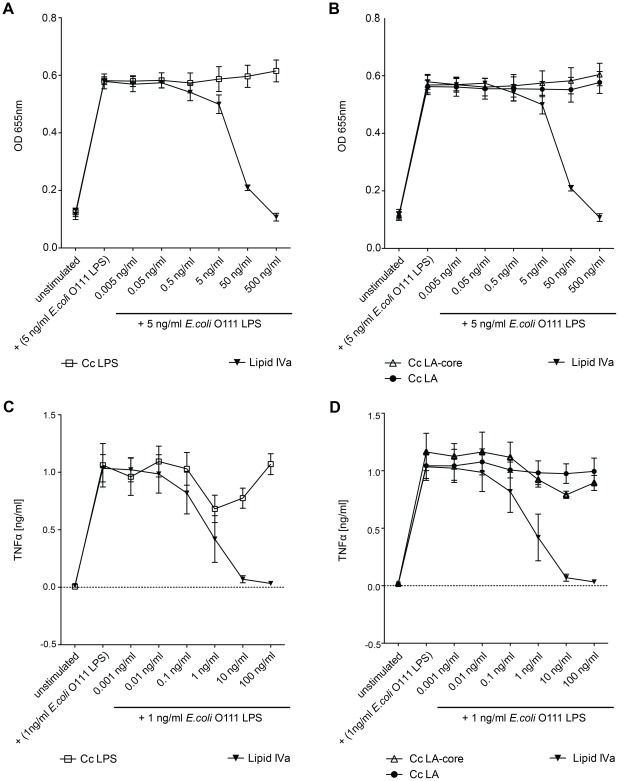
Antagonistic activity of *C. canimorsus* (Cc) LPS, lipid A (LA) or LA-core on the action of *E. coli* O111 LPS. (A–B) HEKBlue human TLR4 cells were preincubated for 3 h with purified lipid A, LA-core or LPS samples at the concentration indicated. Then the cells were stimulated with 5 ng/ml *E. coli* O111 LPS for further 20–24 h and TLR4 dependent NFκB activation was measured. (C–D) Human THP-1 macrophages were preincubated for 3 h with purified lipid A, LA-core or LPS samples at the concentration indicated. Then the cells were stimulated with 1 ng/ml *E. coli* O111 LPS for further 20 h and TNFα release was measured. Data were combined from n = 3 independent experiments, error bars indicated are standard error of the mean.

*C. canimorsus* LPS, lipid A or LA-core were further tested for a possible antagonistic activity on the action of *E. coli* O111 LPS using HEKBlue human TLR4 cells. The cells were preincubated for 3 h with various concentrations of purified *C. canimorsus* lipid A, LA-core or LPS samples, then stimulated with 5 ng/ml *E. coli* O111 LPS for further 20–24 h and the TLR4 dependent NFκB activation was measured. *C. canimorsus* LPS, LA-core and lipid A appeared to be no antagonist of *E. coli* O111 LPS binding to human TLR4, in contrast to the tetra-acylated antagonist lipid IV_A_ (Fig. 7 A and B). In a second assay, human THP-1 macrophages were preincubated for 3 h with purified *C. canimorsus* lipid A, LA-core or LPS samples at the concentration indicated. Then the THP-1 cells were stimulated with 1 ng/ml *E. coli* O111 LPS for further 20 h and TNFα release was measured. *C. canimorsus* lipid A exhibited no antagonism to *E. coli* O111 LPS binding to human TLR4 (Fig. 7 D). Again lipid IV_A_ showed the expected antagonism (Fig. 7 C and D). Dependent on the assay no antagonism or a very weak antagonism of *C. canimorsus* LPS was observed. This is in agreement with the notion of a partial agonist [43], which includes a certain degree of antagonism at sub-agonist concentration.

All tested lipid A and LA-core fractions exhibited no activity towards human TLR2, as tested by HEK293 cells overexpressing human TLR2/MD-2 and a secreted reporter (Fig. S5). This proves that the stimulation of HEKBlue human TLR4 cells with *C. canimorsus* lipid A-core observed is only due to activation of TLR4.

## Discussion

We showed here that *C. canimorsus* has a penta-acylated lipid A, a feature often correlated to low endotoxicity [13], [25]. In addition, the ester-bound 4′ phosphate is lacking. This structural feature is known to reduce the endotoxic activity by a factor of ∼100 [13], which can now be better explained based on the recent data obtained with x-ray crystallography on the TLR4/MD-2/LPS complex [21]. In this complex, phosphate groups of lipid A play a crucial role. The 4′ phosphate is thought to bind to positively charged amino acids in the LRR of TLR4 (Arg_264_, Lys_362_) as well as to MD-2 (Ser_118_ and Lys_58_) in a well-defined manner. This ionic interaction seems to be critical for the ligand affinity of lipid A, enabling formation of a hexameric (TLR4/MD-2/LPS)_2_ complex necessary for signalling [21]. In the endotoxic lipid A, there is another negatively charged group, 1 phosphate, which binds to positively charged amino acids in the complex, especially in the LRR of both TLR4 and the counter TLR4, called TLR4* (Lys_388_* of TLR4*, Lys_341_, Lys_362_ of TLR4) and also to Arg_122_ of MD-2. In contrast to the 4′ phosphate which binds to two proteins (TLR4 and MD-2), the 1 phosphate is involved in binding to three proteins in the complex (TLR4, TLR4*, and MD-2), suggesting that this group might be even more important for the formation of a stable hexameric (LPS/TLR4/MD-2)_2_ complex, as has been reported [44]. We showed in this work that the lipid A of *C. canimorsus* contains a *P*-Etn group at position 1, thus neutralizing the negative charge of the 1 phosphate group. Therefore, we propose that such modified phosphorylation may exert a “shielding effect” on the negative charge of the phosphate and, hence, can explain why the lipid A of *C. canimorsus* is significantly reduced in its endotoxic activity.

The lipid A structure of *C. canimorsus* is similar to that of the closely genetically related *E. meningoseptica* with respect to the nature and position of the acyl chains [30]. As reported for *E. meningoseptica*, we also found some heterogeneity with respect to the nature of the amino sugar at the non-reducing end in the lipid A backbone, but it was significantly lower (2–5% in *C. canimorsus* compared to ∼30% in *E. meningoseptica*) [30]. It has to be pointed out that this structural modification has no influence on the biological activity of lipid A, as it was shown for *Campylobacter jejuni* [45]. The Etn substitution at position 1 of *C. canimorsus* lipid A is however not present in *E. meningoseptica* [30]. One might thus expect that the lipid A of *C. canimorsus* is less endotoxic than that of *E. meningoseptica*. To confirm this suggestion a comparative study of lipid A of both species must be carried out. Since the genus *Capnocytophaga* belongs to the *Bacteroidetes* phylum [46], it is also not surprising that the structure of lipid A from *C. canimorsus* shares some important traits involved in specific TLR4 and MD-2 binding with the structure of *Bacteroides fragilis* lipid A, which we determined earlier [47]. In particular, the lipid A from both bacteria are (3+2) penta-acylated, lack the 4′ phosphate and share *iso*-branched and linear acyl chains, including *i*15:0, 16:0(3-OH), and *i*17:0(3-OH).

In agreement with its structural specifics, *C. canimorsus* lipid A was shown here to exhibit a very low activity towards human TLR4. *C. canimorsus* LPS and LA-core are 100- respectively 1000- fold less active than *E. coli* O111 LPS towards human TLR4, which reminds the activity of the closely related lipid A of *E. meningoseptica* [30].

The data obtained with human TLR4 may seem to contradict previous findings that whole heat killed *C. canimorsus* bacteria do not stimulate human TLR4 [9]. However, in that early study, only one concentration of bacterial lysate was used and compared to purified *E. coli* LPS. From the results presented here, we know that below a certain concentration, pure *C. canimorsus* LPS is weakly active and the threshold concentration for endotoxicity is higher than that of *E. coli* LPS. Thus, the *C. canimorsus* extracts used in previous experiments may have contained LPS in insufficient concentrations. In contrast to what was shown in *Capnocytophaga ochracea* [48], *C. canimorsus* LPS and lipid A were found not to antagonize the action of *E. coli* LPS on human TLR4.

The endotoxicity of the *C. canimorsus* LPS is probably reduced to the level, which is tolerable in the dog's mouth. We found *C. canimorsus* LPS was even slightly less active towards canine than human TLR4 in comparison to *E. coli* LPS. This reduced inflammatory potential might benefit colonization of the dog's mouth. This reduced endotoxicity may probably as well explain why the disease in humans often begins with mild symptoms [2], [6], [49] and finally progresses to severe septicemia with shock and intravascular coagulation. The higher threshold concentration for endotoxicity of *C. canimorsus* LPS is in line with an initial immune evasion. Nevertheless, at high concentrations it reaches an activation comparable to the highly active *E. coli* LPS, which might contribute substanitally to the septic shock observed in patients suffering from *C. canimorsus* infections. Features of the LPS could therefore account for initial evasion of *C. canimorsus* from the host immune system, while the same LPS might later on induce the endotoxic shock when present at higher concentration.

**Figure 8:**
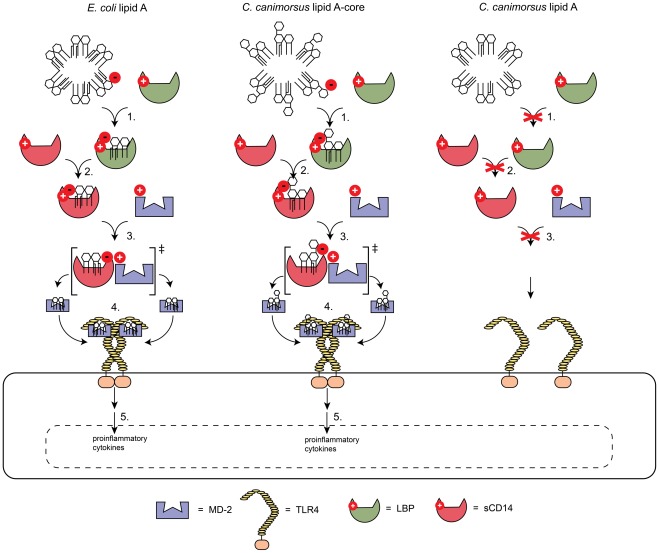
Proposed model for the implication of the LPS core or the 4′ phosphate in enabling the binding to MD-2. Ionic interactions or hydrogen bonds involving the 4′ phosphate or the Kdo carboxy group in LPS lacking a 4′ phosphate enable the binding of lipid A to either LBP (1.), soluble CD14 (sCD14) (2.) or via an intermediate state to MD-2 (3.). Dependent on the type of lipid A bound to MD-2 this leads to TLR4 multimerization (4.), a downstream signaling cascade and finally release of proinflammatory cytokines (5.).

*E. coli* lipid A and O111 LPS exhibit a 10- to 100-fold difference in endotoxicity and similar findings were made for *P. gingivalis* or *Proteus mirabilis* [27], [28]. The lipid A from *E. meningoseptica* also shows only minor differences in TLR4 activation to its LPS [30]. In contrast, we found that *C. canimorsus* lipid A was around 20,000 fold less endotoxic than the LA-core, even higher when compared on a molar basis, suggesting an important role of the core-oligosaccharide in TLR4/MD-2 binding and activation. This indicates the importance of the LPS core for TLR4 activation in the case of *C. canimorsus*, which has a lipid A devoid of a net negative charge. The *C. canimorsus* LPS core exhibits only one unshielded negative charge, on the carboxylic oxygen of Kdo. The negative charged carboxyl-group of Kdo in the *C. canimorsus* core could therefore directly participate in TLR4 or MD-2 binding, besides the reported inner core interactions with TLR4/MD-2 [21]. We found that the MD-2 binding ability of *C. canimorsus* lipid A is strongly reduced compared to the LA-core and we could exclude that changes in solubility were the reason for the differences observed. This finding could explain the difference in endotoxicity, as a lipid A not properly bound to MD-2 cannot activate TLR4. It seems as if the *C. canimorsus* LPS core interacts with CD14, LBP or MD-2 and thus enables the binding to MD-2. By molecular modeling *C. canimorsus* lipid A was predicted to bind MD-2 in a very similar way as *E. coli* lipid A and the calculated binding energy for the two complexes was similar. As the energetic state of the final complex would therefore be stable and favorable in the case of *C. canimorsus* lipid A, we propose that the interactions of the LPS core with MD-2 (or LBP/CD14) preceed the final lipid A – MD-2 binding, rather than only stabilizing it. In our model, summarized in Fig. 8, we suggest an intermediate state in which the lipid A in the case of *E. coli* or the core in the case of *C. canimorsus* form ionic interactions or hydrogen bonds with MD-2 allowing the lipid A – MD-2 complex to form at all. However, we could not rule out a direct role of the LPS-core in binding to CD14 or LBP. To our knowledge, this is the first reported example of the core-oligosaccharide changing dramatically the endotoxicity of lipid A.

## Materials and Methods

### Chemicals

13:0(3-OH) was purchased from Larodan, Malmö, Sveden and 2,3 diamino-2,3-dideoxy-d-glucose (2× HCl) from United States Biochemical Corporation, Cleveland, OH, USA. All other chemicals, solvents and reagents were of highest purity commercially available. *E. coli* O111 LPS was purchased from Sigma-Aldrich, lipidIV_A_ from PeptaNova. *E. coli* F515 lipid A (hexa- and penta-acyl) was purified as described [50], [51]. The analysis and isolation of *C. canimorsus* LA-core will be described elsewhere (Zähringer et al., manuscript in preparation). Purchased reagents were resolved according to manufacturer's instructions. Aliquots of lipid IV_A_ were kept at −80°C.

### Isolation of LPS

*C. canimorsus* bacteria were harvested from 600 blood plates in phosphate buffered saline (PBS) and washed with distilled water, ethanol (300 ml) and acetone (300 ml), followed each time by centrifugation at 18,000× *g* for 30 min. Bacteria were air dried and resuspended in PBS containing 1% phenol for killing and storage in the deep freezer prior to LPS extraction. Cells were washed with ethanol, acetone and diethyl ether (each 1 L) under stirring (1 h, room temperature). After centrifugation cells were dried on air to give 11.2 g. For the isolation of LPS, *C. canimorsus* 5 bacteria were extracted by phenol-water [52]. The LPS was identified in the water phase, which also contained a large amount of an unknown glucan polymer separated by repeated ultracentrifugation (100,000× *g*, 4 h, 4°C, 3 times). The glucan was further analyzed (U. Zähringer and S. Ittig, manuscript in preparation) and the LPS identified in the sediment. The crude LPS preparation was further subjected to RNAse/DNAse treatment (30 mg, Sigma) for 24 h at room temperature followed by Proteinase K digestion (30 mg, 16 h, room temp.) and dialysis (2 days, 4°C), and lyophilization. The yield of enzyme-treated LPS related to bacterial dry mass was 70 mg (0.6%).

### Isolation of lipid A

Lipid A was prepared from *C. canimorsus* 5 (25 mg) LPS by hydrolysis with 2% AcOH (4 ml) at 100°C until precipitation of lipid A (2–8 h). The sediment was extracted three times with a water-chloroform mixture (10 ml) and the organic phase was concentrated to dryness under a stream of nitrogen to give 17.7 mg of crude lipid A. The lipid A was purified by reversed phase HPLC as described elsewhere [53] with the following modifications: an Abimed-Gilson HPLC system equipped with a Kromasil C18 column (5 µm, 100 Å, 10×250 mm, MZ-Analysentechnik) was used. Crude lipid A samples (2–5 mg) were suspended in 0.4 mL solvent A and the mixture was sonicated. A 0.1 M EDTA-sodium salt solution (100 µl, pH 7.0) was added forming a bi-phasic mixture which was vortexed and injected directly onto the column. Samples were eluted using a gradient that consisted of methanol-chloroform-water (57∶12∶31, v/v/v) with 10 mM NaOAc as mobile phase A and chloroform-methanol (70.2∶29.8, v/v) with 50 mM NaOAc as mobile phase B. The initial solvent consisted of 2% B which was maintained for 20 min after injection, followed by a linear three step gradient raising from 2 to 17% B (20–50 min), 17 to 27% B (50–85 min), and 27 to 100% B (85–165 min). The solvent was held at 100% B for 12 min and re-equilibrated 10 min with 2% B and hold for additional 20 min before the next injection. The flow rate for preparative runs was 2 ml/min (∼80 bar) using a splitter (∼1∶35) between the evaporative light-scattering detector (ELSD) and fraction collector. The smaller part of the eluate was split to a Sedex model 75C ELSD (S.E.D.E.R.E., France) equipped with a low-flow nebulizer. The major part was collected by a fraction collector in 1 min intervals (∼2 ml each). Nitrogen (purity 99.996%) was used as gas to nebulize the post column flow stream at 3.5 bar into the detector at 50°C setting the photomultiplier gain to 9. The detector signal was transferred to the Gilson HPLC Chemstation (Trilution LC, version 2.1, Gilson) for detection and integration of the ELSD signal.

### GLC and GLC-MS analyses

Sugar and fatty acid derivatives were analysed by gas-liquid chromatography (GLC) on a Hewlett-Packard HP 5890 Series chromatograph equipped with a 30-m fused-silica SPB-5 column (Supelco) using a temperature gradient 150°C (3 min)→320°C at 5°/min. GLC-MS was performed on a 5975 inert XL Mass Selective Detector (Agilent Technologies) equipped with a 30-m HP-5MS column (Hewlett-Packard) under the same chromatographic conditions as in GLC.

### ESI-MS analysis

Analyses of lipid A were performed in negative and positive ion modes on a high resolution Fourier transform ion cyclotron resonance mass spectrometer, FT ICR-MS (Apex Qe, Bruker Daltonics, Billerica, MA, USA), equipped with a 7 T superconducting magnet and an Apollo dual electrospray-ionization (ESI)/Matrix-assisted laser desorption ionization (MALDI) ion source. Data were recorded in broadband mode with 512 K data sampling rate. The mass scale was calibrated externally by using compounds of known structure. For the negative ion mode samples (ca. 10 ng/µl) were dissolved in a 50∶50∶0.001 (v/v/v) mixture of 2-propanol/water/triethylamine (pH∼8.5). For the positive ion mode samples, a 50∶50∶0.03 (v/v/v) mixture of 2-propanol/water/30 mM ammonium acetate adjusted with acetic acid to pH 4.5 was used. The samples were sprayed at a flow rate of 2 µL/min. The capillary entrance voltage was set to 3.8 kV and the drying gas temperature to 150°C. The mass numbers given refer to that of the monoisotopic ion peak. For MS/MS in the positive ion-mode small amounts of TEN were added to the sample preparation to obtain the [M+TEN+H]^+^ adduct ions [32] which were selected for collision induced decay (CID) in the collision cell infrared multiphoton dissociation (IRMPD) within the ion cycIotron resonance (ICR) cell.

### NMR spectroscopy

Lipid A samples (1–3 mg) were exchanged twice with deuterated solvents [chloroform-*d*_1_/methanol-*d*_4_ 1∶1 (v/v), Deutero GmbH, Kastellaun, Germany] and evaporated to dryness under a stream of nitrogen. Samples were dissolved in 180 µl chloroform-*d*_1_/methanol-*d*_4_/D_2_O 40∶10∶1 (v/v/v, 99.96%) and analyzed in 3 mm NMR tubes (Deutero). ^1^H-, ^13^C-, and ^31^P-NMR spectra were recorded at 700.7 MHz (^1^H) on an Avance III spectrometer equipped with a QXI-cryoprobe (Bruker, Germany) at 300 K. Determination of NH-proton signals was performed in chloroform-*d*_1_(99.96%)/methanol/H_2_O 40∶10∶1 without exchange in deuterated solvents. Chemical shifts were referenced to internal chloroform (δ_H_ 7.260, δ_C_ 77.0). ^31^P NMR spectra were referenced to external aq. 85% H_3_PO_4_ (δ_P_ 0.0). Bruker software Topspin 3.0 was used to acquire and process the NMR data. A mixing time of 100 ms and 200 ms was used in TOCSY and ROESY experiments, respectively.

### Compositional analyses

Quantification of GlcN, GalN (internal standard) and GlcN3N by GLC and GLC-MS was done after strong acid hydrolysis of 0.5 mg lipid A in 4 M HCl (16 h, 100°C), followed by acetylation (*N*-acetylation) in pyridine/acetic acid anhydride (10 min, 85°C), reduction (NaBH_4_) and per-*O*-acetylation. The response factor of the per-*O*-acetylated GlcNAc-ol, GalNAc-ol, and GlcNAc3NAc-ol derivatives, necessary for the quantification of GlcN3N by GLC, was determined in addition by external calibration with synthetic reference sugars. Etn, GlcN, GlcN3N and their corresponding phosphates (GlcN-*P* and Etn-*P*), were determined from the hydrolysate by reversed phase HPLC using the Pico-tag method and pre-column derivatization with phenylisothiocyanate according to the supplier's instructions (Waters, USA). Quantification of total phosphate was carried out by the ascorbic acid method [54]. For analysis of ester- and amide-linked acyl chains, the lipid A was isolated from LPS (1 mg) by mild acid hydrolysis (0.5 mL, 1% AcOH, 100°C, 2 h), centrifuged and the lipid A sediment was separated into two aliquots and lyophilized. Ester-linked acyl chains were liberated from the first aliquot by treatment with 0.05 M NaOMe in water-free methanol (0.5 mL) at 37°C for 1 h. The mixture was dried under a stream of nitrogen and acidified (M HCl) prior to extraction with chloroform. The free acyl chains were converted into methyl esters by treatment with diazomethane and hydroxylated acyl chains were trimethylsilylated with *N,O*-bis(trimethylsilyl)trifluoroacetamide for 4 h at 65°C [55]. The acyl chain derivatives were quantified by GLC-MS using the corresponding derivatives of 17:0 (50 µg) and 13:0(3-OH) (50 µg, Larodan, Malmö, Sweden) as internal standards for the calibration of the response factor of non-hydroxylated and hydroxylated acyl chains, respectively. For analysis of total acyl chains, the second aliquot was subjected to a combined acid/alkaline hydrolysis as described [56]. Briefly, acyl chains were liberated from the lipid A by strong acid hydrolysis (4 M HCl, 100°C, 21 h) and extracted three times with water/chloroform (0.5 mL each). The organic phase containing the *N*- and *O*-linked acyl chains was treated with diazomethane, trimethylsilylated and quantified as described above.

### Bacterial strains and growth conditions

The strains used in this study are listed in Table S2. *E. coli* strains were grown in LB broth at 37°C. *C. canimorsus* 5 [9] was routinely grown on Heart Infusion Agar (HIA; Difco) supplemented with 5% sheep blood (Oxoid) for 2 days at 37°C in presence of 5% CO_2_. Bacteria were harvested by scraping colonies off the agar surface, washed and resuspended in PBS. Selective agents were added at the following concentrations: erythromycin, 10 mg/ml; cefoxitin, 10 mg/ml; gentamicin, 20 mg/ml; ampicillin, 100 mg/ml.

### Human TLR4 activation assay

HEK293 stably expressing human TLR4, MD-2, CD14 and a secreted NFκB dependent reporter were purchased from InvivoGen (HEKBlue hTLR4). Growth conditions and endotoxicity assay were as recommended by InvivoGen. Briefly, desired amount of LPS or lipid A were placed in a total volume of 20 µl (diluted in PBS) an added a flat-bottom 96-well plate (BD Falcon). 25000 HEKBlue hTLR4 cells in 180 µl were then added and the plate was incubated for 20–24 h at 37°C and 5% CO_2_. If the antagonistic activity of a compound on the action of *E. coli* O111 LPS was assayed, the compound was added in a total volume of 10 µl (diluted in PBS), 25000 HEKBlue hTLR4 cells in 180 µl were added and the plate was incubated for 3 h at 37°C and 5% CO_2_. Then the cells were stimulated with 5 ng/ml *E. coli* O111 LPS and the plate was incubated as above. Detection followed the QUANTI-Blue protocol (InvivoGen). 20 µl of challenged cells were incubated with 180 µl detection reagent (QUANTI-Blue, InvivoGen). Plates were incubated at 37°C and 5% CO_2_ and colour developed was measured at 655 nm using a spectrophotometer (BioRad). If needed the *C. canimorsus* lipid A stock solution (1 mg/ml) was supplemented with 0.1% v/v TEN or 50% v/v DMSO and sonicated for some minutes just before the assay. The TEN containing lipid A stock solution was further diluted in a 0.1% TEN solution to keep the TEN concentration constant in all samples. Due to the high concentration of DMSO used, this lipid A stock solution was further diluted with PBS. As a control the same amount of TEN or DMSO has been added to *E. coli* O111 LPS samples tested in the same assay. DMSO concentration in 50 µg/ml and 5 µg/ml were found to affect physiological test conditions. These data have therefore been excluded from the figure.

### TNFα release by human THP-1 cells

Human THP-1 monocytes (ATCC TIB-202) were cultured as recommended by the American Type Culture Collection (RPMI 1640 medium complemented with 10% v/v heat-inactivated fetal bovine serum, 2 mM L-Glutamine). Monocytes were seeded at 1.5×10^5^ cells/ml in 24 well-plates (BD Falcon) in growth medium containing 10^−7^ M PMA (Sigma-Aldrich). For differentiation and attachment the cells were incubated for 48 h at 37°C and 5% CO_2_ and then washed with growth medium and fresh PMA-free medium was added. After further incubation for >1 h the cells were challenged with the indicated amount of LPS or lipid A in a total volume of 20 µl (diluted in PBS). After 20 h of incubation the supernatants were harvested and immediately analyzed for TNFα by an ELISA. ELISA was performed in accordance with the manufacturers instructions (BD OptEIA). If an antagonist of *E. coli* O111 LPS was assayed, the compound was added in a total volume of 10 µl (diluted in PBS) to the THP-1 cells and the plates were incubate for 3 h at 37°C and 5% CO_2_. Then the cells were stimulated with 1 ng/ml *E. coli* O111 LPS and the plate was incubated for 20 h at 37°C and 5% CO_2_.

### IL-6 release by canine DH82 macrophages

Canine DH82 macrophages (ATCC CRL-10389) were cultured in DMEM supplemented with 15% v/v heat-inactivated fetal bovine serum, 2 mM L-Glutamine and non-essential amino acids (Sigma-Aldrich) in a humidified incubator at 37°C and 5% CO_2_. Cells were seeded at 1×10^5^ cells/ml in 24 well-plates (BD Falcon). The cells were incubated for 24 h at 37°C and 5% CO_2_, before being challenged with the indicated amount of LPS in a total volume of 10 µl (diluted in PBS). After 14 h of incubation the supernatants were harvested and immediately analyzed for content of IL-6 by an ELISA. ELISA was performed in accordance with the manufacturer's instructions (R&D Systems, DY1609).

### LPS biotinylation

Biotinylation of *E. coli* O111 LPS (Sigma-Aldrich) was performed as described previously [57] using biotin-LC-hydrazide (Pierce, Rockford, IL). To verify that the biotinylation did not affect the functionality of the LPS, *E. coli* LPS-Biotin was assayed for endotoxicity with the HEKBlue human TLR4 cell line (Data not shown). Biotinylation reduced the endotoxic potential at low concentrations, but only slightly at concentrations used in the MD-2 binding assay.

### Human MD-2 binding assay

MD-2 binding assays was performed as described [57], [58]. HEK293 cells were transfected using Fugene6 (Roche, 3∶2 protocol) with a plasmid (kind gift of K. Miyake and C. Kirschning) encoding human MD-2 with a C-terminal Flag-His-tag (pEFBOS-hMD2-Flag-His) [15]. The medium was exchanged 3–8 h post transfection with fresh growth medium. The cells were incubated for 48 h and the supernatant was harvested and pooled. Fresh FCS was added to the hMD-2 supernatant (7.5% v/v) as a source of CD14 and LBP. For each binding reaction, 4 ml of hMD-2 supernatant were combined with 250 ng, 500 ng, 1 µg, 2 µg, 5 µg or 10 µg of the competitor, incubated at room temperature and gently rocked for 30 min. If needed the *C. canimorsus* lipid A stock solution (1 mg/ml) was supplemented with 0.1% v/v TEN or 50% v/v DMSO and sonicated for some minutes just before addition to the hMD-2 supernatant. 1 µg of biotinylated *E. coli* O111 LPS was added and the supernatant was further incubated for 3–4 h at room temperature. Biotinylated LPS–hMD-2 complexes or single biotinylated LPS were captured by addition of 120 µl (total volume) streptavidin-agarose beads (IBA) per sample. The beads were previously prepared by washing them three times with a buffer (100 mM Tris, 150 mM NaCl, pH 8.0). For binding, the supernatants containing the beads were incubated overnight on a rotator at 4°C. Agarose beads were pelleted by centrifuging for 30 s at 5000× *g* and 4°C and washed three times with PBS containing 0.5% Tween 20. The beads were finally resuspended in 60 µl SDS-loading dye (without dithiothreitol) and boiled for 5 min at 95°C. The protein content in the sample was analyzed by non-reducing, denaturing 4–12% Tris-glycine Polyacrylamide gels (Invitrogen) or 4–15% Tris-glycine Polyacrylamide gels (BioRad) and then transferred to polyvinylidene fluorid (PVDF) membrane (ImmobilonP, Millipore). Membranes were probed using monoclonal anti-Flag antibody (Sigma-Aldrich) according to the manufacturer's instructions using ECL-Plus reagent (GE Healthcare).

### Genome annotation

*Blast*-p search tool [59] against the *C. canimorsus* 5 genome [5] was used. Search sequences were obtained from the National Center for Biotechnology Information. All available *Bacteroidetes*-group sequences were used as search if available, but standard *E. coli* sequences have always been included. The highest scoring subjects over all the searches have been annotated as corresponding enzymes. Difficulties in annotation were only observed for *lpxE*. *lpxE* search was based on *lpxF* and/or *lpxE* sequences from *P. gingivalis* [38], *F. novicida* [60], *R. etli* [40], *H. pylori* [37], [41] and on all available *Bacteroidetes*-group *pgpB* sequences. Three *lpxE/F* candidates have been found in the *C. canimorsus 5* genome (*Ccan* 16960, *Ccan* 14540 and *Ccan* 6070). All candidates have been deleted and only deletion of *Ccan* 16960 affected endotoxicity (data not shown). Since this gene is encoded in an operon with the predicted *eptA* and since the same operon structure (*lpxE-eptA*) has been identified in *H. pylori* [37] *Ccan*16960 was annotated as *lpxE*.

### Molecular modeling

The MD-2 - *E. coli* LPS complex (PDB code 3FXI) [21] was used to construct models for the MD-2 - *E. coli* lipid A and for the MD-2 – *C. canimorsus* Lipid A. The modeling of the lipid A moieties was performed using the VMD [61] program and the *leap* module of the AMBER11 [62] suite of programs. To investigate the time-dependent properties of the two MD-2 – lipid A complexes, the constructed systems were subjected to molecular dynamics simulations [63] in the framework of a classical molecular mechanics [64] (MM) description. MM parameters from the Glycam06 [65], [66] force field were adapted to describe the acyl chains and the sugar moieties, while the Amber99SB [67], [68] force field was employed for the MD-2 protein. Advanced methods based on quantum chemistry were employed to obtain the missing parameters of the ester linkages and hydroxyl groups on the acyl chain C2 atoms, the branching at the bottom of the *C. canimorsus* acyls, the phosphate/*P*-Etn groups and the GlcN3N′ moiety. Bonding parameters were obtained by performing relaxed potential energy scans [69] (bonds, angles, dihedrals), while charges were calculated on the optimized geometries of selected capped fragments. All the scan and geometry optimizations were conducted at the RI-MP2/def2-TZVP [70], [71], [72] level using the COBRAMM [73] suite of programs efficiently linking the ORCA2.8 [74] (wave-function calculation) and the GAUSSIAN09 [75] (optimization/scan driver) programs. Charges were calculated according to the RESP procedure at the HF/6-31G*//MP2/def2-TZVP. Both MD-2 – lipid A complexes were embedded in a 6.5×6.5×6.5 nm^3^ box of TIP3P [76] water molecules and the appropriate number of Na^+^ and Cl^−^ ions were added to neutralize the systems charge. The systems were relaxed (conjugate gradient geometry optimization) to remove clashes before stating molecular dynamics simulations. The systems were both heated to 300 K in the NVT (constant particle number, volume, temperature) ensemble for 500 ps and then equilibrated in the NPT (constant particle number, pressure, temperature) until relevant structural parameters (density, RMSD on the protein Cα) were found to be stable (1 ns). Statistics were then performed on trajectories collected from 10 ns long simulations of the equilibrated systems. All molecular dynamics calculations were performed with the sander module of the AMBER11 package; bonds involving H atoms were constrained using the SHAKE algorithm [77] to allow for using a time step of 2 fs. Pressure was controlled *via* a simple Berendsen weak coupling approach [78], while a Langevin thermostat (collision frequency set to 3 ps^−1^) was used to enforce the desired temperature. Molecular dynamics trajectories were analyzed using the VMD software, the *ptraj* module of the AMBER11 suite and the ProDy [79] package. A set of 300 snapshots of the equilibrated trajectories was subjected to further analysis to quantify the binding energy between MD-2 and each of the two lipid A moieties. Both the MM-PBSA and MM-GBSA approaches [80] were used to calculate the MD-2 – lipid A binding energy, while a full interaction energy decomposition [81], [82] was performed using the cheaper MM-GBSA method; the *MMPBSA.MPI* module of AMBER11 was used to perform the binding free energy calculations, while a locally developed software was used to process, analyze and plot the results.

### Western-blot quantification

Quantification was performed using MultiGauge software (Fujifilm).

## Supporting Information

Figure S1 HPLC elution profile of the lipid A from *C. canimorsus* 5. HPLC elution profiles of the semi-preparative fractionation of the lipid A from wild type *C. canimorsus* 5 (2.1 mg). Peak Nr. 2 (124.3 min) represents the intact lipid A and peak Nr. 5 (131.9 min) the 1-dephosphorylated lipid A (LA without *P*-Etn) as determined by ESI MS. The other peaks belong to lipid A with slightly modified fatty acids composition (Table 2). For HPLC conditions see Materials and Methods.
(TIF)

Figure S2 Negative mode ESI mass spectrum of lipid A from *C. canimorsus* indicating heterogeneity in the length of fatty acids (-CH_2_-, Δ*m/z* = 14 u) as also shown in Table 2.
(TIF)

Figure S3 CID-MS/MS (positive mode) of lipid A from *C. canimorsus* showing the B-fragment (non-reducing end) obtained from the parent ion [M+TEN+H]^+^ [*m/z* 1819.3]. The abundant B-fragment ion is consistent with a GlcN3N carrying two primary fatty acids [16:0(3-OH) and *i*17:0(3-OH)] in amide linkage and one (*i*15:0) in ester linkage forming an acyloxyacyl residue [*i*17:0(3-*O*(*i*15:0)] and proves the hybrid backbone (GlcN3N′-GlcN) to be the major one (>95%) and the distribution of the fatty acids to be 3+2.
(TIF)

Figure S4 ^1^H,^31^P-HMQC (top) and ^1^H,^31^P-HMQC-TOCSY (bottom) spectra (700 MHz) of lipid A in chloroform-methanol-water (20∶10∶1, v/v/v) at 27°C. The ^31^P NMR spectrum and the corresponding part of the ^1^H NMR spectrum are displayed along the F1 and F2 axes, respectively. Numerals refer to atoms in sugar and acyl chain residues denoted by letters as shown in Supplementary Table 1 and Fig. S2.
(TIF)

Figure S5 Activation of human TLR2 with *C. canimorsus* (Cc) or *E. coli* lipid A (LA) or LA-core preparations. Indicated concentrations of purified lipid A or LA-core samples were assayed for TLR2 dependent NFκB activation with HEKBlue human TLR2 cells. The triacylated lipopetide Pam_3_CSK_4_ was used a positive control. Data were combined from n = 3 independent experiments, error bars indicated are standard error of the mean.
(TIF)

Table S1 ^1^H (700 MHz) and ^13^C (176.2 MHz) NMR data of the lipid A from *C. canimorsus* (CDCl_3_/MeOD/D_2_O, 40∶10∶1, v/v/v). Chemical shifts are referenced to internal CDCl_3_ (δ_H_ 7.26, δ_C_ 77.0) at 27°C. For the assignment of the individual acyl chains see Fig. 2.
(XLS)

Table S2 Bacterial strains used in this study.
(DOC)

Text S1 Supplementary methods.
(DOC)
