# Supplementary figures and images for: The Lipopolysaccharide from Capnocytophaga canimorsus Reveals an Unexpected Role of the Core-Oligosaccharide in MD-2 Binding
Source: PLoS Pathog. 2012 May 3;8(5):e1002667. doi: 10.1371/journal.ppat.1002667 (PMC3342949; doi:10.1371/journal.ppat.1002667)

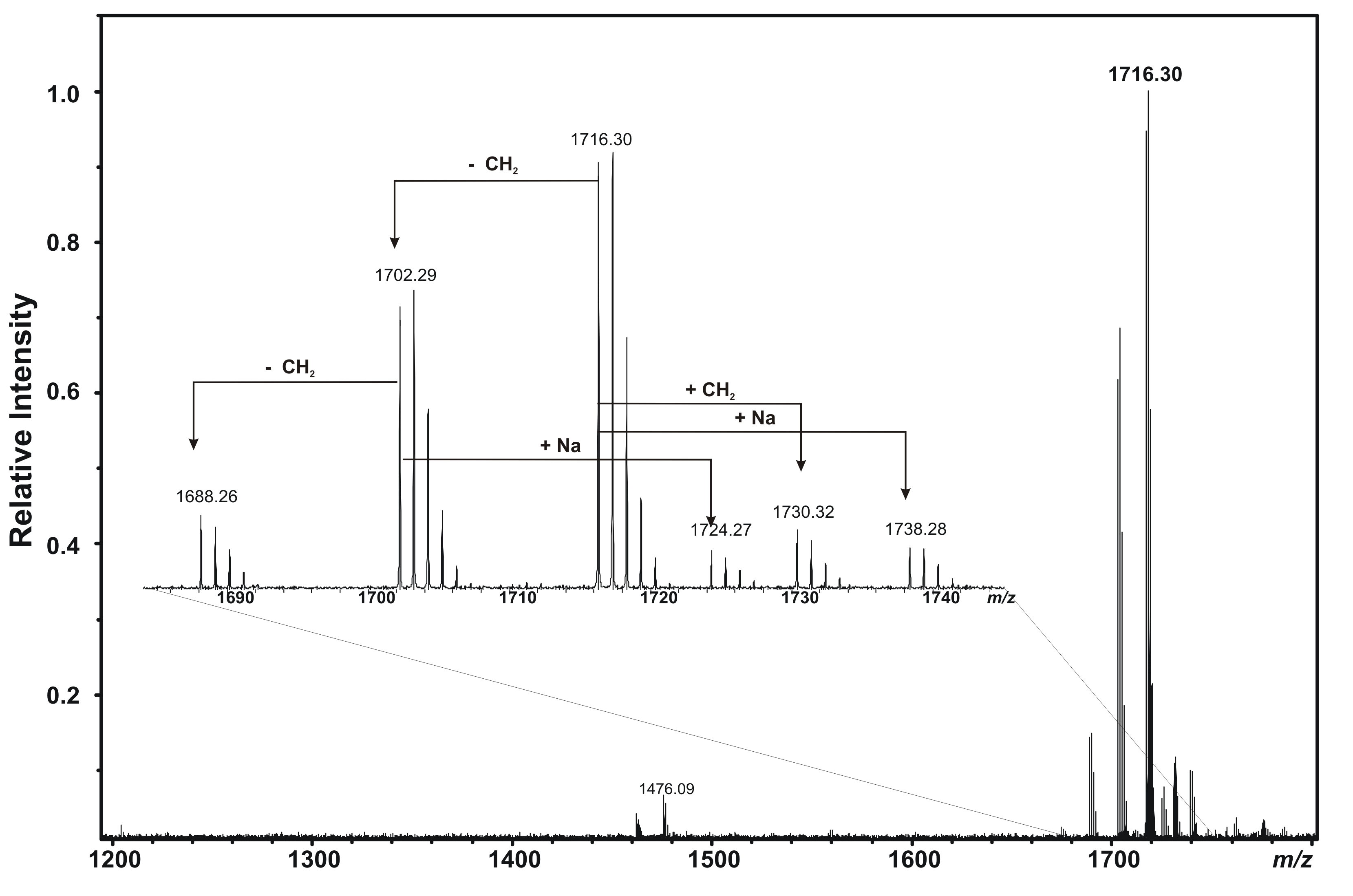

Supplement: Figure S2 — Negative mode ESI mass spectrum of lipid A from C. canimorsus indicating heterogeneity in the length of fatty acids (-CH2-, Δm/z = 14 u) as also shown in Table 2. (TIF) [file ppat.1002667.s002.tif]

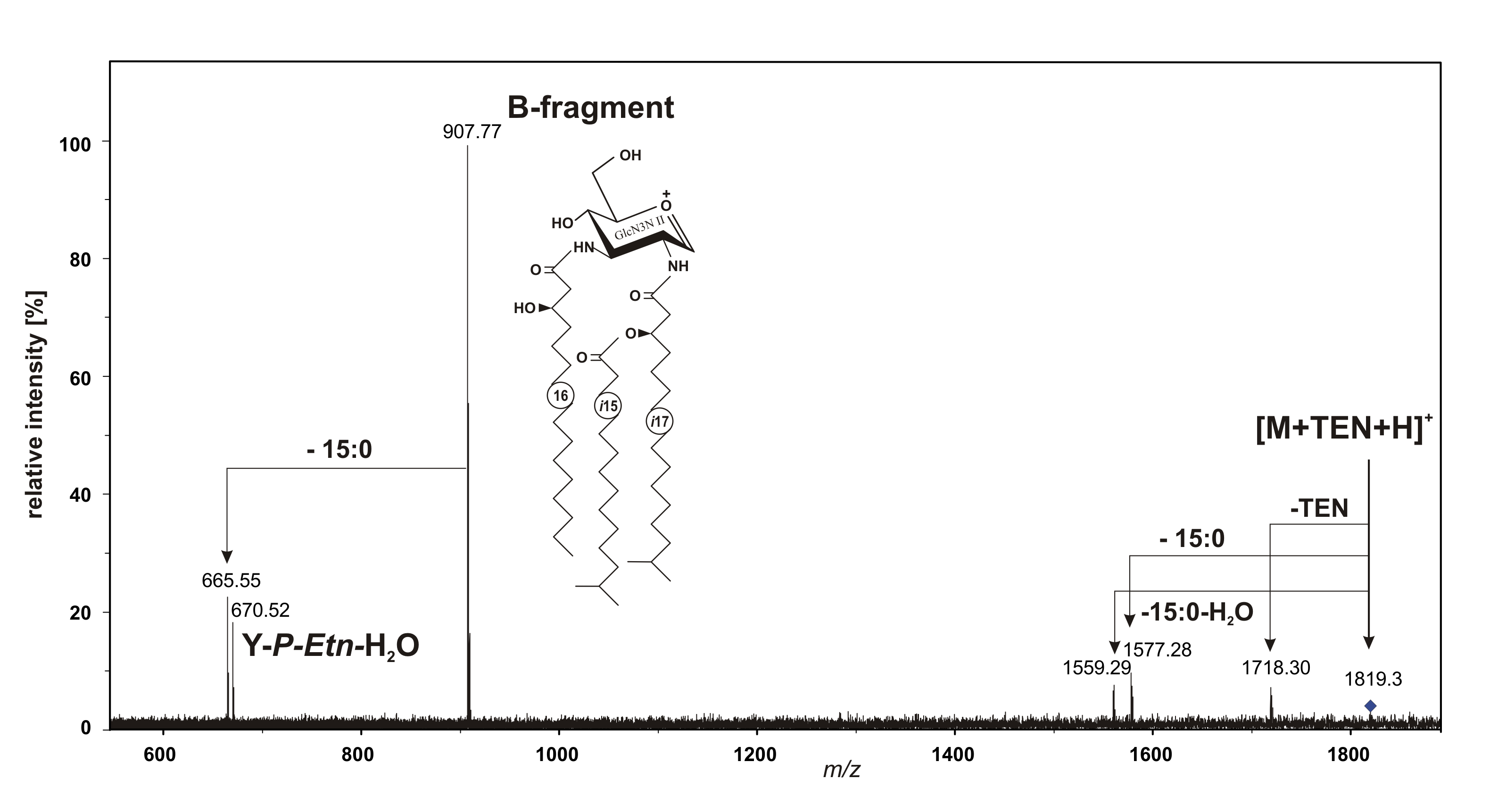

Supplement: Figure S3 — CID-MS/MS (positive mode) of lipid A from C. canimorsus showing the B-fragment (non-reducing end) obtained from the parent ion [M+TEN+H]+ [m/z 1819.3]. The abundant B-fragment ion is consistent with a GlcN3N carrying two primary fatty acids [16:0(3-OH) and i17:0(3-OH)] in amide linkage and one (i15:0) in ester linkage forming an acyloxyacyl residue [i17:0(3-O(i15:0)] and proves the hybrid backbone (GlcN3N′-GlcN) to be the major one (>95%) and the distribution of the fatty acids to be 3+2. (TIF) [file ppat.1002667.s003.tif]

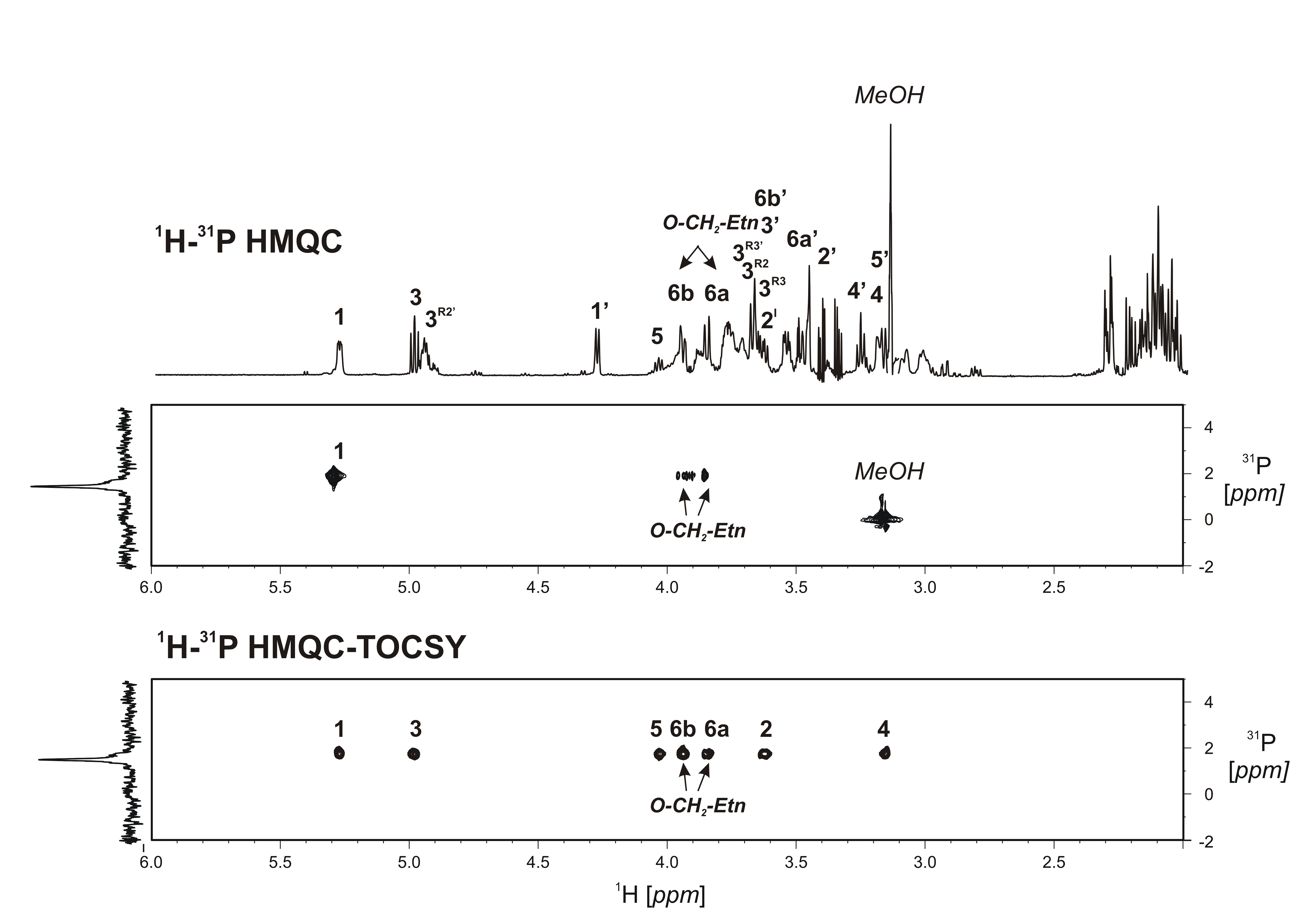

Supplement: Figure S4 — 1H,31P-HMQC (top) and 1H,31P-HMQC-TOCSY (bottom) spectra (700 MHz) of lipid A in chloroform-methanol-water (20∶10∶1, v/v/v) at 27°C. The 31P NMR spectrum and the corresponding part of the 1H NMR spectrum are displayed along the F1 and F2 axes, respectively. Numerals refer to atoms in sugar and acyl chain residues denoted by letters as shown in Supplementary Table 1 and Fig. S2. (TIF) [file ppat.1002667.s004.tif]

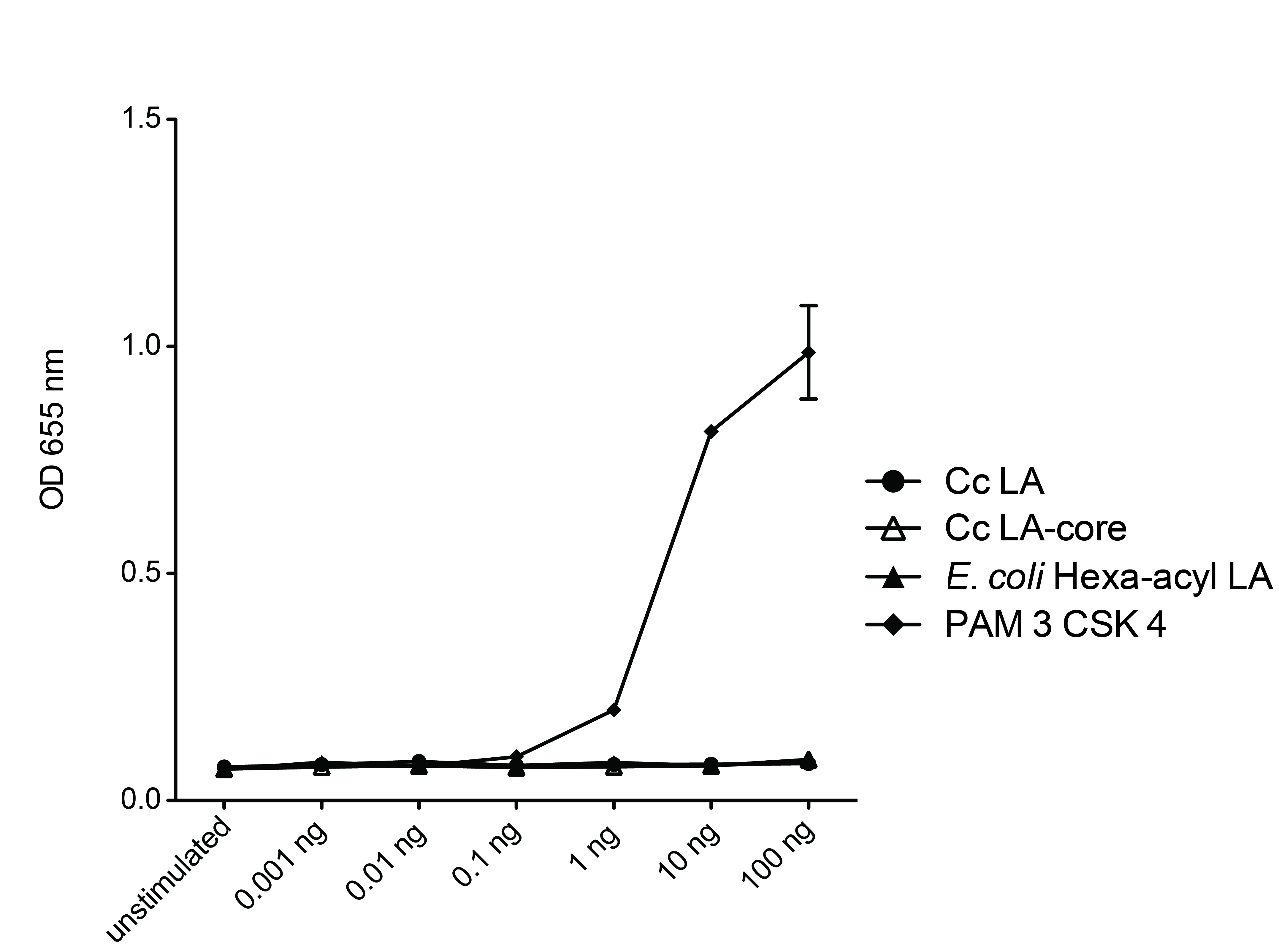

Supplement: Figure S5 — Activation of human TLR2 with C. canimorsus (Cc) or E. coli lipid A (LA) or LA-core preparations. Indicated concentrations of purified lipid A or LA-core samples were assayed for TLR2 dependent NFκB activation with HEKBlue human TLR2 cells. The triacylated lipopetide Pam3CSK4 was used a positive control. Data were combined from n = 3 independent experiments, error bars indicated are standard error of the mean. (TIF) [file ppat.1002667.s005.tif]
