# Supplementary material for: The Lipopolysaccharide from Capnocytophaga canimorsus Reveals an Unexpected Role of the Core-Oligosaccharide in MD-2 Binding
Source: PLoS Pathog. 2012 May 3;8(5):e1002667. doi: 10.1371/journal.ppat.1002667 (PMC3342949; doi:10.1371/journal.ppat.1002667)
Supplement: Table S2 — Bacterial strains used in this study. (DOC) [file ppat.1002667.s007.doc]

| Bacterial strains | Description or genotype | Reference or source |
| --- | --- | --- |
| *E. coli* |  |  |
| F515 | Deep rough mutant | [51] |
| Top10 | F*- mcrA* Δ*(mrr-hsd*RMS*-mcr*BC*) φ80lac*ZΔM*15*  Δ*lac*X74 *rec*A1 *ara*D139 Δ*(araleu)*7697 *gal*U *gal*K  *rps*L*, end*A1 *nup*G*.* Sm^r^ | Invitrogen |
| *C. canimorsus* |  |  |
| *Cc5* | Human fatal septicemia after dog bite 1995 | [9], [5] |
