# Supplementary material for: The Lipopolysaccharide from Capnocytophaga canimorsus Reveals an Unexpected Role of the Core-Oligosaccharide in MD-2 Binding
Source: PLoS Pathog. 2012 May 3;8(5):e1002667. doi: 10.1371/journal.ppat.1002667 (PMC3342949; doi:10.1371/journal.ppat.1002667)
Supplement: Text S1 — Supplementary methods. (DOC) [file ppat.1002667.s008.doc]

**Supplementary methods**

**Human TLR2 activation assay.** HEK293 transfected with human TLR2 and CD14 were purchased from InvivoGen (HEKBlue hTLR2). Growth conditions and agonist assay were performed in accordance with recommendations of InvivoGen. Briefly, desired amount of the stimulus in a total volume of 20 μl (diluted in PBS) were added to a well of a flat-bottom 96-well plate (BD Falcon). 25000 HEKBlue hTLR2 cells in 180ul were added and the plate was incubated for 20-24h at 37°C and 5% CO_2_. Detection followed the QUANTI-Blue protocol (InvivoGen). 20 μl of challenged cells were incubated with 180 μl detection reagent (QUANTI-Blue, InvivoGen). Plates were incubated at 37°C and 5% CO_2_ and developed colour was measured using a spectrophotometer (BioRad) at 655nm. Pam_3_CSK_4_ (InvivoGen) was used as positive control.
